# Aseptische Meningitis infolge intravenöser Immunglobulin‐Therapie (IVIG) bei therapierefraktärem Pyoderma gangraenosum: Aseptic meningitis due to intravenous immunoglobulin therapy (IVIG) in therapy‐refractory pyoderma gangraenosum

**DOI:** 10.1111/ddg.70263x

**Published:** 2026-11-10

**Authors:** Katharina A. Kälber, Knut Schäkel, Alexander Enk, Julia K. Winkler

**Affiliations:** ^1^ Universitäts‐Hautklinik Heidelberg Ruprecht‐Karls Universität Heidelberg Heidelberg Deutschland

Sehr geehrte Herausgeber,

Wir berichten über eine 33‐jährige Patientin, die erstmals 2017 großflächige livid‐erythematöse, teils nekrotische und stark schmerzhafte Hautveränderungen an beiden Ober‐ und Unterschenkeln entwickelte. Bei der Erstvorstellung in unserer Hautklinik 2019 zeigten sich ausgedehnte Ulzerationen mit Nekrosen an beiden Oberschenkeln (Abbildung 1). An relevanten Vorerkrankungen war seit 2010 eine Colitis ulcerosa (CU) bekannt. Initiale Therapieversuche der CU umfassten Azathioprin, Ciclosporin A und Infliximab. Bei unzureichender Besserung der CU erfolgte im Oktober 2010 eine totale Kolektomie mit Pouchversorgung und späterer Anlage eines Ileostoma. Klinisch und histologisch waren die Ulzerationen vereinbar mit einem Pyoderma gangraenosum (PG); der PARACELSUS Score betrug 11 (Abbildung 2). Das PG ist eine seltene neutrophile Dermatose,^1^ mit Prävalenzen zwischen 0,3 und 1/100.000, das weibliche Geschlecht ist überrepräsentiert. PG kann assoziiert sein mit CU, Morbus Crohn, chronischer Polyarthritis, monoklonaler Gammopathie, Lymphomen und systemischen Vaskulitiden.^2^

**ABBILDUNG 1:**
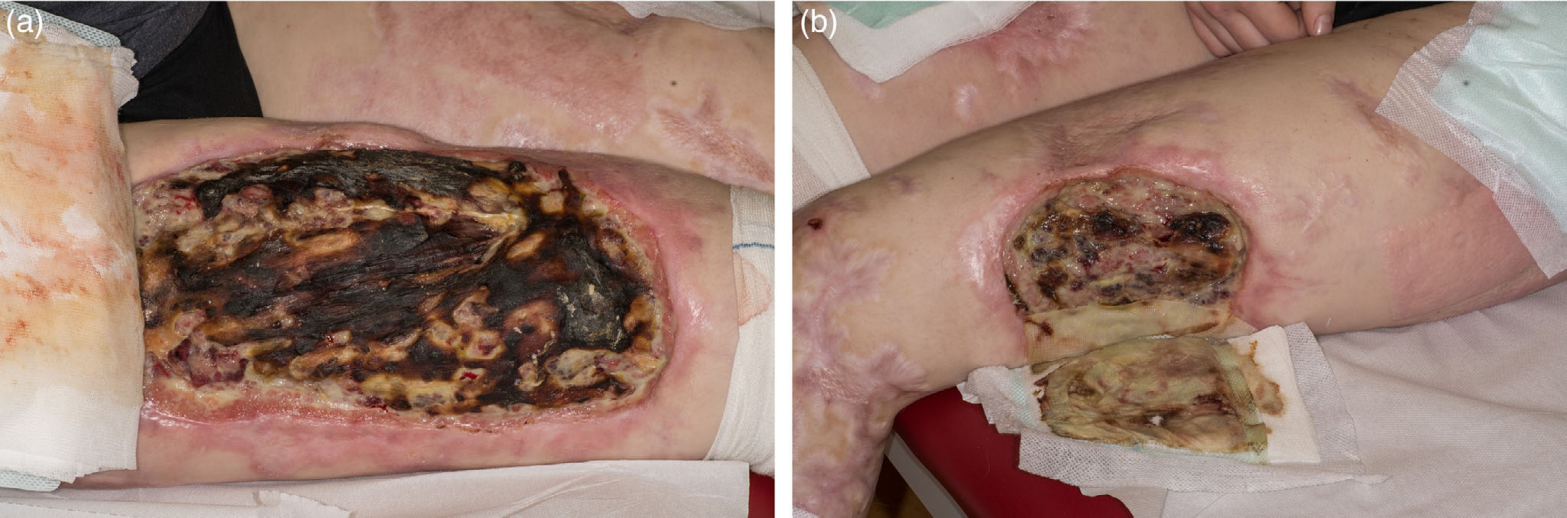
(a) Klinischer Befund im Rahmen der Erstvorstellung in unserer Ambulanz 2019. Am rechten Oberschenkel zeigte sich eine circa 20 x 30 cm messende, großflächige Ulzeration mit Nekrosen, die teils bis zur Muskulatur reichten. Zudem fanden sich multiple Narben an den Ober‐ und Unterschenkeln. (b) Klinischer Befund im Rahmen der Erstvorstellung in unserer Ambulanz 2019. Am linken Oberschenkel zeigte sich eine circa 10x18 cm messende Ulzeration mit Nekrosen und Fibrinbelägen. Zudem kamen multiple Narben nach Spalthautentnahme zur Darstellung.

**ABBILDUNG 2:**
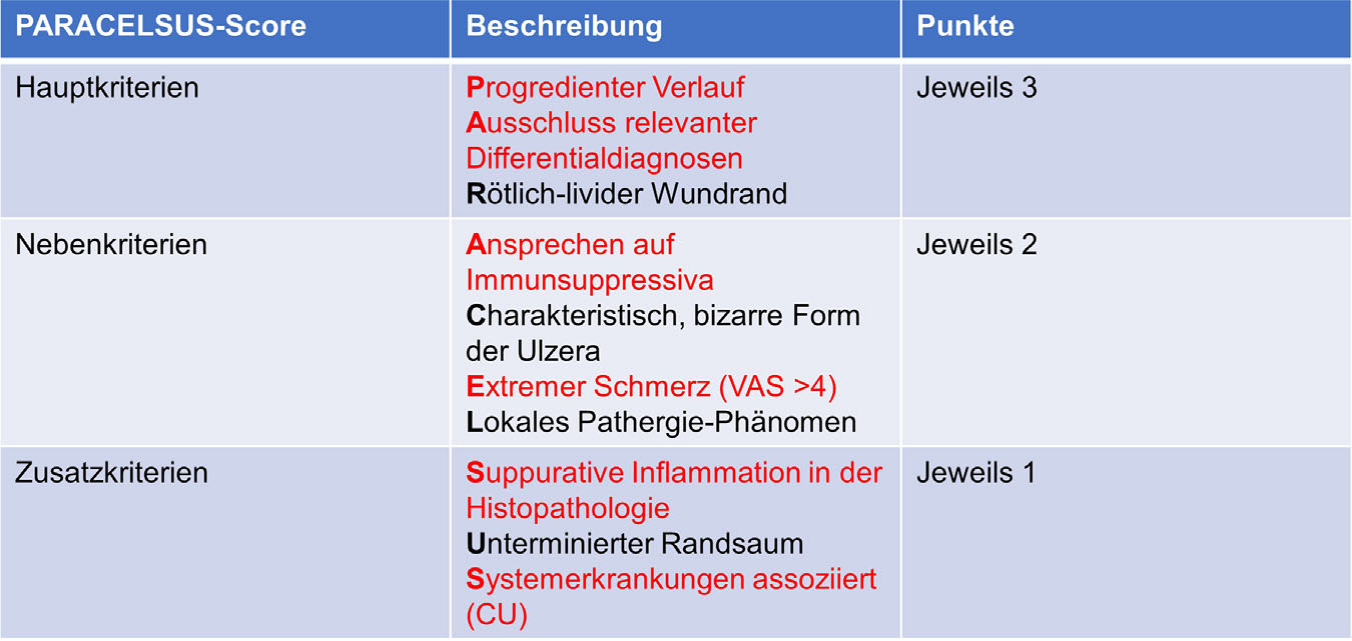
PARACELSUS Score. Die bei unserer Patientin zutreffenden Kriterien wurden rot markiert. Auswertung: Ab 10 Punkte = Pyoderma gangraenosum sehr wahrscheinlich < 10 Punkte = Pyoderma gangraenosum sehr unwahrscheinlich.

Bei unserer Patientin wurde differenzialdiagnostisch auch eine Dermatitis artefacta diskutiert. Das ausgeprägte Schmerzempfinden, die histopathologischen Befunde, die Assoziation mit der CU sowie das teilweise Ansprechen auf Immunsuppressiva sprachen jedoch gegen eine primär artifizielle Genese.

Unter Azathioprin, systemischem Prednisolon und Adalimumab (Tabelle 1) kam es initial zu partiellen Befundbesserungen, jedoch nicht zu anhaltender Remission mit vollständigem Wundverschluss. Fehlendes nachhaltiges Therapieansprechen unter immunsuppressiven Therapien ist bei PG möglich. Vor der Erstvorstellung in unserer Ambulanz erfolgten mehrere Debridements, Vakuumtherapien (*Vacuum Assisted Closure*, VAC) und Spalthauttransplantationen, die nur kurzfristig zur Besserung führten, gefolgt von erneuter Verschlechterung.

**TABELLE 1:** Übersicht über die Systemtherapien von 2010 – 2025.

Zeitraum	Therapie	Kommentar/Anlass
07/2010	Azathioprin 150 mg/d	Abgesetzt bei unzureichender Wirkung auf Colitis ulcerosa (CU)
2010	Ciclosporin A^*^	Unzureichende Besserung der CU
08 – 09/2010	Infliximab 5 mg/kg Körpergewicht alle 2 Wochen	Keine Besserung der CU
11/2017	Prednisolon^*^ + Azathioprin^*^	Therapie des Pyoderma gangraenosum (PG)
05/2018 – 07/2018	Adalimumab 40 mg alle 2 Wochen	Keine nachhaltige klinische Besserung
03/2018 – 04/2019	Adalimumab 40 mg + Prednisolon‐Stoßtherapien 20–40 mg/d	Keine Remission
07/2019 – 10/2019	Ciclosporin A^*^ + Tacrolimus 8 mg/d	Stationäre Aufnahme wegen Anämie und Wundinfektion
03/2020 – 06/2020	Infliximab 5 mg/kg Körpergewicht alle 2 Wochen	Gute klinische Wirkung, aber allergische Reaktionen
06/2020 – 09/2020	Infliximab‐Desensibilisierung	Trotz Prämedikation Flush/Übelkeit
09/2020 – 11/2020	Omalizumab 300 mg + Infliximab 5 mg/kg Körpergewicht	Abbruch bei Hypotonie und Tachykardie nach 5. Desensibilisierung
01/2021	IVIG hochdosiert 2 g/kg Körpergewicht	Aseptische Meningitis, anamnestisch Abheilung des PG
01/2025	Prednisolon + subkutane Beriglobin‐Gaben in Eigenregie^*^	Unzureichende Besserung des PG bei Rezidiv seit 11/2024
03/2025	Upadacitinib 15 mg/d + Mycophenolsäure 1440 mg/d	Unzureichendes Ansprechen
05/2025	Prednisolon 50 mg/d + Upadacitinib 15 mg/d + Mycophenolsäure 1440 mg/d	Unzureichendes Ansprechen
05/2025	IVIG hochdosiert 2 g/kg Körpergewicht	Abbruch bei Wiederauftreten der aseptischen Meningitis
07/2025	Mycophenolsäure 720 mg/d + Tacrolimus 4 mg/d	PG größenprogredient
08/2025	Mycophenolsäure (Monotherapie) 720 mg/d	Verschlechterung Tacrolimus wegen Nierenfunktionsverschlechterung
12/2025	Anakinra 100 mg/d	Besserung der Ulzerationen

* Diese Therapien erfolgten extern. Eine Aussage zu den genauen Dosierungen konnte nicht angegeben werden.

Im März 2020 wurde eine Therapie mit Infliximab eingeleitet. Im Rahmen der ersten Infliximab‐Gaben kam es zu Übelkeit, Flush‐Symptomatik mit Urtikaria und Erbrechen, die sich unter Glukokortikoidgabe besserten. In der Prick‐Testung konnte eine Typ‐1 Sensibilisierung auf Infliximab nachgewiesen werden. Bei klinischem Ansprechen des PG auf die Infliximab‐Therapie erfolgte eine Desensibilisierung.^3^ Es kam jedoch trotz Prämedikation, langsamer Aufdosierung und zusätzlicher Gabe von Omalizumab wiederholt zu Flush, Emesis, einem Gefühl der Todesangst und Dyspnoe mit Tachykardie und Hypotonie. Auf Grund der Anaphylaxie musste die Infliximab‐Desensibilisierung trotz therapeutischer Wirksamkeit beendet werden.

Anschließend wurde eine Therapie mit hochdosierten intravenösen Immunglobulinen (IVIG 2 g/kg Körpergewicht) eingeleitet. Nach der ersten Gabe entwickelte die Patientin stärkste Kopfschmerzen mit Lichtempfindlichkeit, Erbrechen und Fieber bis zu 39°C. Laborchemisch zeigten sich geringgradig erhöhte Infektwerte. Im Liquor fand sich eine erhöhte Zellzahl mit 42 Zellen/µl bei sonst unauffälliger Liquorchemie. Die zytologische Untersuchung ergab ein lymphozytäres Zellbild. Ergänzend fanden sich keine Hinweise auf eine Schrankenstörung oder intrathekale Immunglobulinsynthese. Die Patientin wurde notfallmäßig stationär aufgenommen und empirisch mit Ceftriaxon und Aciclovir behandelt. Es ergab sich klinisch und in der Elektroenzephalographie (EEG) kein Anhalt auf eine Enzephalitis. Die Herpes simplex (HSV) und Varizella zoster Virus (VZV) PCR im Liquor waren negativ. Es wurde eine aseptische Meningitis nach IVIG‐Gabe diagnostiziert. Hierzu passend war der unmittelbare zeitliche Zusammenhang zwischen IVIG‐Gabe und Beginn der Symptomatik innerhalb von zwei Tagen mit rascher Besserung nach Absetzen der IVIG.

**TABELLE 2:** Übersicht wesentlicher IVIG‐Nebenwirkungen und deren Management. Die Häufigkeiten wurden gemäß der folgenden Konvention bestimmt: sehr häufig (≥ 1/10); häufig (≥ 1/100, < 1/10); gelegentlich (≥ 1/1.000, < 1/100); selten (≥ 1/10.000, < 1/1.000) und sehr selten (< 1/10.000).

Nebenwirkungen	Symptome	Häufigkeit	Management
Infusionsreaktionen	Kopfschmerzen, Übelkeit, Flush	Sehr häufig	Infusionsrate reduzieren, Prämedikation
Kopfschmerzen	Spannungs‐ oder migräneartige Schmerzen	Sehr häufig	Hydration, Analgetika, Infusionsrate senken
Fieber / Schüttelfrost	Temperaturanstieg nach Infusion	Häufig	Antipyretika, Infusionspause
Hypertonie / Blutdruckschwankungen	Anstieg oder Schwankung des Blutdrucks	Häufig	Vitalzeichenmonitoring, Infusion anpassen
Tachykardie	Erhöhte Herzfrequenz	Häufig	Beobachtung, Infusionsrate reduzieren
Allergische Reaktionen	Urtikaria, Pruritus, Bronchospasmus, Dyspnoe	Gelegentlich	Infusion stoppen, Antihistaminikum, ggf. Steroide
Thromboembolische Ereignisse	Tiefe Venenthrombose (TVT^*^), Lungenembolie, Schlaganfall, Myokardinfarkt	Gelegentlich bis selten	Risikofaktoren minimieren, ausreichende Hydration, Thromboseprophylaxe
Aseptische Meningitis	Starke Kopfschmerzen, Photophobie	Selten	IVIG absetzen, Analgetika, Flüssigkeit, Ausschluss infektiöser Ursachen
Akutes Nierenversagen	Kreatininanstieg	Selten	Hydration, Präparatwechsel, Nierenfunktion überwachen
Anaphylaxie	Hypotonie, Bronchospasmus	Sehr selten	Notfalltherapie: Adrenalin, Steroide, Antihistaminika, Infusion stoppen
Transfusionsassoziierte akute Lungeninsuffizienz (TRALI)^**^	Hypoxie, pulmonales Infiltrat	Sehr selten	Sofort Infusion stoppen, intensivmedizinische Unterstützung

* Eine TVT ist ein Blutgerinnsel (Thrombus) in einer tiefen Vene, meist in den Beinen, die zu Schwellung, Schmerzen, Rötung und zu einer Lungenarterienembolie führen kann.

** Die transfusionsassoziierte akute Lungeninsuffizienz (TRALI) ist eine seltene, aber schwere Reaktion auf IVIG und Bluttransfusionen, die sich durch plötzliche Atemnot, Sauerstoffmangel und Lungenödeme innerhalb von bis zu sechs Stunden nach der Transfusion äußert und lebensbedrohlich sein kann, wobei Antikörper im Spenderblut die Lungenkapillaren schädigen.

Die Behandlung der aseptischen Meningitis nach IVIG basiert auf supportiven Maßnahmen wie Analgetika‐Gabe und Flüssigkeitsmanagement.^4^ In den meisten Fällen klingen die Symptome innerhalb von ein bis zwei Wochen ohne bleibende Schäden ab.

Die aseptische Meningitis unter hochdosierter IVIG‐Therapie ist eine seltene, aber bereits berichtete Nebenwirkung^5^ (Tabelle 2). Der genaue Mechanismus ist nicht vollständig geklärt und es werden unterschiedliche Pathomechanismen diskutiert. IVIG lösen eine Immunantwort aus, die die Freisetzung von Zytokinen fördert, welche eine Entzündung im zentralen Nervensystem (ZNS) verursachen können. In einem Fallbericht wurden die Zytokine und Chemokine im Liquor von einem Patienten mit aseptischer Meningitis nach IVIG und Patienten mit viraler Meningitis untersucht.^6^ Hier zeigten sich bei der IVIG‐assoziierten Meningitis erhöhte Spiegel des *Monocyte Chemoattractant Protein‐1* (MCP‐1) im Liquor. Es wurde postuliert, dass der Anstieg von MCP‐1 im Liquor über die Aktivierung von Monozyten zur Entwicklung einer aseptischen Meningitis führen könnte. Zudem können IVIG Immunkomplexe bilden, die das Immunsystem aktivieren und so zur Pathogenese einer aseptischen Meningitis beitragen könnten. Einige IVIG‐Präparate enthalten als Stabilisatoren Saccharose und/oder Maltose, die eine osmotische Wirkung haben und eine lokal toxische oder reizende Wirkung auf die Meningen entfalten können.^7^

Typische Symptome der aseptischen Meningitis sind Kopfschmerzen, Fieber, Nackensteifigkeit bis hin zu neurologischen Defiziten, die innerhalb von ein bis sieben Tagen nach IVIG Gabe auftreten.^8^ Zur Vorbeugung gegen aseptische Meningitis nach IVIG werden langsame Infusionsgeschwindigkeit und ausreichende Flüssigkeitszufuhr empfohlen; hierfür gibt es jedoch keine belastbare Evidenz aus prospektiven Studien.^9^

Anamnestisch kam es laut Patientin nach der einmaligen IVIG‐Gabe zur vollständigen Abheilung der Ulzerationen. Da sie sich in den folgenden drei Jahren nicht erneut vorstellte, konnte dieser Verlauf nicht objektiviert werden. Im November 2024 wurde im Rahmen einer beidseitigen Lungenarterienembolie die Erstdiagnose einer chronisch thromboembolischen pulmonalen Hypertonie (CTEPH) gestellt und eine pulmonale Endarteriektomie durchgeführt. Gleichzeitig entwickelte die Patientin ein etwa 35×12 cm großes Rezidiv des Pyoderma gangraenosum lateral am rechten Oberschenkel. Bei unzureichendem Ansprechen auf systemische Glukokortikoide, Mycophenolsäure und Upadacitinib erfolgte nach kritischer Risiko‐Nutzen Abwägung der Entschluss zur erneuten Gabe intravenöser IVIG. Trotz langsamer Infusionsgeschwindigkeit entwickelte die Patientin am dritten Tag der IVIG‐Gabe Übelkeit, Erbrechen, stärkste Kopfschmerzen (VAS 9/10) und Fieber von 39,5°C. Es war erneut eine stationäre Behandlung bei aseptischer Meningitis erforderlich. Aufgrund des zeitlichen Zusammenhangs und der Antikoagulation bei Zustand nach multiplen thromboembolischen Ereignissen in der Vorgeschichte erfolgte keine erneute Lumbalpunktion. Die Patientin erhielt eine supportive Therapie in Form von Flüssigkeit, Kalium‐Substitution bei rezidivierender Hypokaliämie sowie eine analgetische und antipyretische Therapie. Darunter besserte sich die Symptomatik rasch und die Patientin konnte stabilisiert entlassen werden. Es bildeten sich anschließend einzelne Epithelinseln im Bereich der Ulzeration am rechten Oberschenkel, es kam jedoch nicht zu durchgreifender Befundbesserung des PG.

Im interdisziplinären Entzündungsboard wurde ein kurzzeitiger Therapieversuch mit hochpotenten topischen Glukokortikoiden unter einem okklusiven Verband empfohlen, da die Patientin erneut eine Spalthauttransplantation durchführen lassen wollte.

Zudem wurde auch eine weitere Abklärung im Hinblick auf Genmutationen, die das Immun‐ oder Komplementsystem betreffen könnten, angeraten. In der Literatur werden zur Behandlung des PG Therapieversuche mit IL‐(12/)23‐, IL‐36‐Antikörper und dem IFX‐1 Komplement C5a Inhibitor diskutiert.^10^ Aktuell werden mehrere klinische Studien zur Behandlung des Pyoderma gangrenosum durchgeführt. Hierzu zählt unter anderem eine randomisierte, placebo‐kontrollierte Studie mit dem Anti‐IL‐36‐Rezeptor‐Antikörper Spesolimab (NCT06624670). Von einer Behandlung mit einem IL‐17‐Antikörper wurde bei der Patientin aufgrund der bekannten CU abgesehen.

Im weiteren Verlauf erfolgte eine plastische Deckung der ausgedehnten Ulzeration. Parallel wurde eine Therapie mit dem IL‐1‐Rezeptorantagonisten Anakinra eingeleitet. Hierunter zeigte sich aktuell ein gebesserter Befund.

IVIG sind eine etablierte, effiziente und in der Regel sehr gut verträgliche Therapie für viele Autoimmundermatosen, die eine Reduktion des Bedarfs an Glukokortikoiden und anderen Immunsuppressiva ermöglichen.^11^ Die meisten Nebenwirkungen sind mild und symptomatisch zu behandeln, jedoch können auch schwere Nebenwirkungen wie thromboembolische Ereignisse, Anaphylaxien und sehr selten auch eine aseptische Meningitis auftreten.

## DANKSAGUNG

Open access Veröffentlichung ermöglicht und organisiert durch Projekt DEAL.

## INTERESSENKONFLIKT

Keiner.
